# Cholecystogastric fistula: A case report and review of the literature

**DOI:** 10.1016/j.ijscr.2024.110141

**Published:** 2024-08-14

**Authors:** Emad Aljohani, Matar Awadalla, Wala Abdelkarim, Abdulkarim Alkadrou

**Affiliations:** a Associate Professor, Department of Surgery, College of Medicine, Prince Sattam Bin Abdulaziz University, Saudi Arabia; b General Surgery Consultant, Department of Surgery, Dr. Suliman Alhabib Medical Group, Riyadh, Saudi Arabia; c General Surgery Specialist, Department of Surgery, Dr. Suliman Alhabib Medical Group, Riyadh, Saudi Arabia; d General Surgery Resident, Department of Surgery, Dr. Suliman Alhabib Medical Group, Riyadh, Saudi Arabia

**Keywords:** Cholecystogastric fistula, Cholelithiasis, Laparoscopic cholecystectomy

## Abstract

**Introduction:**

Cholecystogastric fistula is an abnormal communication between the gallbladder and the stomach, it is a rare complication of chronic cholethiasis. Preoperative diagnosis is difficult as patients often present with non-specific symptoms.

**Case presentation:**

A 63-year-old female presented to the outpatient clinic with symptomatic cholelithiasis. Physical examination was unremarkable. Laboratory investigations, including complete blood count (CBC) and liver function test (LFT), were within normal limits. Upper abdominal ultrasound revealed hepatomegaly and gallbladder contraction with multiple gallstones. Intraoperative exploration during laparoscopic cholecystectomy revealed adhesions with cholecystogastric fistula, necessitating meticulous dissection, fistula excision, and primary closure.

Postoperatively, the patient recovered uneventfully, with a negative methylene blue leak test allowing early oral intake. Discharged home in stable condition, subsequent follow-up showed resolution of symptoms, and histopathological examination confirmed absence of neoplastic changes.

**Discussion:**

Optimal surgical management of cholecystogastric fistula is debatable, laparoscopic surgery have led to improved outcomes in the management of these cases. Utilizing which approach should be determined based on the clinical scenario for each patient and the surgeon experience.

**Conclusion:**

Cholecystogastric fistula is a rare complication of chronic cholethiasis. Preoperative diagnosis requires high index of suspicion. Complete laparoscopic management is safe.

## Highlights

• Cholecystogastric fistula, a rare cholelithiasis complication, is challenging to diagnose due to vague symptoms.
• Meticulous dissection in case of incidental fistula during Laparoscopic cholecystectomy is the key to effective management.
• While optimal surgery is debatable, the laparoscopic approach provides better outcomes, based on clinical scenarios and surgeon expertise

## 1 Introduction

The majority of patients with gallstones are asymptomatic. Only 20 % experience symptoms, and this subgroup is at high risk of developing serious complications varying from biliary colic to severe pancreatisis [1]. Cholecystoenteric fistula is a rare complication of cholethiasis. The duodenum (77 to 90 %) and the hepatic flexure of the colon (8 to 26.5 %) are the most common sites. Cholecystogastric fistula (CGF) accounts for only 2 % of internal biliary fistulas [2] with limited reports in literature.

Patients with cholecystogastric fistula present with non-specific symptoms, CT findings which suggest the diagnosis include two approximated organs with an edematous wall, pericholecystic inflammatory change, a gallstone in the gastrointestinal tract, and direct visualization of the fistula [3]. Previously, laparoscopic approach to manage incidental cholecystogastric fistula was contraindicated. However, it became more feasible nowadays. The work has been reported in line with the SCARE criteria [4].

## 2 Case presentation

A 63-year-old female with no significant medical history or previous abdominal surgeries, presented with a one-year history of recurrent postprandial right upper quadrant pain, accompanied by radiation to the back, as well as nausea and vomiting. Physical examination was unremarkable, with a soft abdomen devoid of tenderness.

Laboratory investigations, including a complete blood count (CBC) and liver function tests (LFT), returned unremarkable results. Notably, the CBC indicated a white blood cell count of 8.5 × 109/L and a hemoglobin level of 12.6 mg/dL. The LFT panel demonstrated values within normal parameters, including alkaline phosphatase (ALP) at 108 U/L, aspartate aminotransferase (AST) at 11 U/L, alanine aminotransferase (ALT) at 9 U/L, direct bilirubin at 2.2 μmol/L, and total bilirubin at 5 μmol/L. Upper abdominal ultrasound revealed hepatomegaly with a liver size measuring 17 cm, accompanied by mild hepatic fatty infiltration. The gallbladder appeared contracted with multiple gallstones, while the common bile duct (CBD) showed normal diameter.

**Fig. 1:**
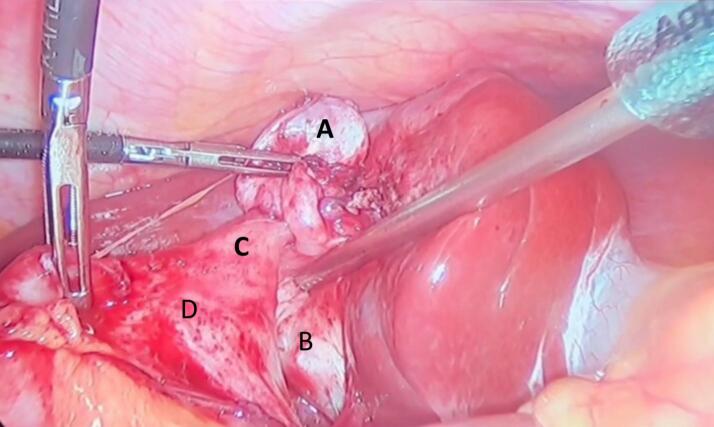
Cholecystogastric fistula attached to the gallbladder body. A: Gallbladder fundus, B: Hartmann's pouch C: pre-pyloric cholecystogastric fistula, D: stomach.

The patient was scheduled for elective laparoscopic cholecystectomy. Intraoperatively, extensive dense adhesions were encountered in the right upper quadrant, involving the gallbladder and stomach, with complete omental adherence to the gallbladder. Meticulous adhesiolysis unveiled a pre-pyloric cholecystogastric fistula (Fig. 1). The fistula tract was meticulously dissected and excised, followed by primary closure of the gastric defect and reinforcement with an omental patch. Subsequently, a complete cholecystectomy was performed, and an abdominal drain was inserted.

Postoperatively, the patient experienced an uncomplicated recovery. A negative methylene blue leak test on postoperative day four allowed for the initiation of oral intake. Discharge home in a stable condition. At the one-week follow-up appointment, the patient remained asymptomatic, and the histopathological examination confirmed the presence of a fistula tract, devoid of neoplastic changes.

## 3 Discussion

CGF is an uncommon complication of chronic cholethiasis and recurrent episodes of inflammation, the fistulous tract forms due to gradual erosion of the gallbladder wall and the wall of the stomach, other reported causes include peptic ulcer, inflammatory bowel disease, and GI malignancy [5]. Clinical diagnosis of CGF is challenging and most cases are discovered intra-operatively, with a mortality rate of 19–24 % [6]. Surgical management remains the mainstay of treatment. Although incidental discovery of CGF during laparoscopic cholecystectomy warranted conversion to open surgery in the past, laparoscopic approach is gaining popularity with the availability of advanced instruments, and the improvement of intracorperal suturing and knotting skills [7].

Many surgeons still choose to convert to an open approach when encountering difficult dissection of dense adhesions. However, the laparoscopic approach is preferred over open surgery due to the enhanced magnification provided by the laparoscope allows for more precise dissection, which is particularly beneficial in complex cases involving dense adhesions and fistula. Complete laparoscopic management of CGF is possible with a conversion rate of 6.3 % [8].

Another area of debate is the one-stage versus two stage surgery. One stage surgery includes: transaction of fistula tract, repair of the gastric fistula site and cholecystectomy in the same setting versus delayed cholecystectomy in the two stage operation [9]. Patient's clinical condition, surgeon's skills and anatomical visibility are factors to be considered when choosing which approach to apply.

Moreover, transection of fistula tract using endostapler illustrated good results in avoiding peritoneal contamination [8]. Dividing the tract after securing either end with ligatures is another option. In line with Nayak et al. our patient also had an incomplete fistula with involvement of the seromuscular layers only, gastric mucosa wasn't penetrated, closure of the diseased gastric site can be achieved by primary suturing, stapler or omental patching [10].

## 4 Conclusion

Cholecystogastric fistula is a rare complication of chronic cholethiasis. Preoperative diagnosis requires high index of suspicion. Complete laparoscopic management is safe with meticulous adhesiolysis and dissection by the experienced surgeon. Closure of the involved gastric site by omental patching is recommended.

## Consent for publication

Written informed consent was obtained from the patient for publication of this case report and accompanying images.

## Ethical approval

This case report did not involve experimental interventions or procedures beyond standard clinical care. Therefore, ethical approval was not deemed necessary as it did not include deviations from routine medical practice.

## Funding

No funding was received for this case report.

## Guarantor

Dr. Emad Aljohani.

## CRediT authorship contribution statement

Dr. Emad Aljohani: Manuscript editing and the case presentation
Dr. Matar Awadalla: Writing the discussion
Dr. Wala Abdelkarim: Writing the literature review
Dr. Abdulkarim Alkadrou: Writing the case presentation.

## Declaration of competing interest

The authors declare that they have no competing interests.
